# Genetic diversity of astroviruses detected in wild aquatic birds in Hong Kong

**DOI:** 10.1186/s12985-024-02423-w

**Published:** 2024-07-07

**Authors:** Daisy Y. M. Ng, Wanying Sun, Thomas H. C. Sit, Christopher J. Brackman, Anne C. N. Tse, Christine H. T. Bui, Amy W. Y. Tang, Andrew N. C. Wong, Andrew T. L. Tsang, Joe C. T. Koo, Samuel M. S. Cheng, Malik Peiris, Alex W. H. Chin, Leo L. M. Poon

**Affiliations:** 1 School of Public Health, LKS Faculty of Medicine, The University of Hong Kong, Hong Kong, China; 2 Agriculture, Fisheries and Conservation Department, Government of the Hong Kong Special Administrative Region, Hong Kong, China; 3 Centre for Immunology and Infection, Hong Kong Science and Technology Park, Hong Kong, China; 4 HKU-Pasteur Research Pole, School of Public Health, LKS Faculty of Medicine, The University of Hong Kong, Hong Kong, China

**Keywords:** Astrovirus, Avastrovirus, Aquatic bird

## Abstract

Wild waterfowl serve as a reservoir of some astroviruses. Fecal samples from wild waterfowl collected at Hong Kong's Marshes were tested using pan-astrovirus reverse transcription-PCR. Positive samples underwent subsequent host identification using DNA barcoding. Based on deduced partial sequences, noteworthy samples from three astrovirus groups (mammalian, avian and unclassified astroviruses) were further analyzed by next-generation sequencing. One sample of *Avastrovirus* 4 clade, MP22-196, had a nearly complete genome identified. The results of ORF2 phylogenetic analysis and genetic distance analysis indicate that *Avastrovirus* 4 is classified as a distinct subclade within *Avastrovirus*. MP22-196 has typical astrovirus genome characteristics. The unique characteristics and potential differences of this genome, compared to other avian astrovirus sequences, involve the identification of a modified sgRNA sequence situated near the ORF2 start codon, which precedes the ORF1b stop codon. Additionally, the 3' UTR of MP22-196 is shorter than other avian astroviruses. This study expands our understanding of the *Avastrovirus* 4 clade.

**Supplementary Information:**

The online version contains supplementary material available at 10.1186/s12985-024-02423-w.

## Introduction

Astroviruses, belonging to the *Astroviridae* family, are non-enveloped viruses with positive-sense single-stranded RNA genomes [1]. It is known to infect a variety of hosts, including avian and mammalian species, and causes asymptomatic to severe disease. Many literatures have documented that astroviruses are closely related to poultry diseases, affecting poultry production and causing considerable economic losses. Disease records associated with avian enteritis include runting-stunting syndrome (RSS), poult enteritis complex or syndrome (PEC/PES), and poult enteritis mortality syndrome (PEMS) [2–7]. Avian nephritis virus (ANV) is associated with nephritis and growth inhibition in chicks [8–10]. Astroviruses have also been found in ducks suffering from viral hepatitis leading to acute death [11]. Chicken astrovirus, goose astrovirus, and duck astrovirus can cause gout disease in poultry [12, 13]. However, many astroviruses cause mild diseases or asymptomatic infections, leading to widespread astrovirus circulation in poultry. In mammals, mild symptoms are common. Human astroviruses are common enteric viruses that cause enteritis and diarrhea in neonates, immunocompromised individuals, and the elderly [1].

Astroviruses can stably circulate in the environment or different hosts for a long time. The virus has been detected in more than 80 host species [14]. According to the 2019 proposal of International Committee on Taxonomy of Viruses (ICTV), astroviruses can be divided into two genera: *Mamastrovirus* and *Avastrovirus*. The former comprises 19 recognized species, while the latter consists of three recognized species, namely *Avastrovirus* 1–3 [15–17]. The genus *Mamastrovirus* is primarily associated with mammals. Avian astroviruses belong to the genus *Avastrovirus* and are the focus of this study. However, a novel mamastrovirus was identified in the European roller, a wild carnivorous bird in Hungary, in recent years. This indicates the possibility of cross-species infection and cross-class astroviruses [18]. There are also studies describing suspected avian astrovirus infection in mammals [19, 20]. Cross-species transmission events indicate that interactions between host species allow astroviruses to evolve and potentially infect new hosts, increasing zoonotic risk [16]. Regular monitoring of the evolution and transmission rates of these viruses can help expand the epidemiological information on astroviruses. Moreover, based on analyses of partial and incomplete virus sequences, the current understanding of astrovirus diversity is likely underestimated.

Hong Kong is situated on the East Asian-Australasian Flyway, one of the nine migratory bird routes worldwide. During the non-breeding season (November to April), numerous migratory birds, including endangered species, gather in Hong Kong's Mai Po wetland (22°29′56″N 114°02′45″E). This area serves as one of the important feeding stations and resting points for these wild waterfowl to spend the winter. Over a decade ago, our team discovered astroviruses in wild bird fecal samples, revealing novel virus diversity [21]. This study aims to build upon our previous research by using pan-astrovirus reverse transcription-PCR to monitor and understand the diversity and spread of astroviruses in migratory birds. By employing current phylogenetic analysis and metagenomic methods, we successfully elucidate *Avastrovirus* clade 4 (*Avastrovirus* 4) which was previously unexplored. This study presents the first near-complete genome of *Avastrovirus* 4, detected in migratory bird fecal samples from the Mai Po Wetland, along with its genomic characterization. These findings contribute to a better understanding of the features and evolution of these viruses.

## Materials and methods

### Sample collection and processing

Fecal swab samples were collected randomly from Mai Po marshes in Hong Kong during the winters of 2018–2019 (*N* = 94) and 2020–2021 (*N* = 94). Additional site visits were organized from November 2022 to April 2023 and 1524 fecal samples were collected randomly. These samples were stored in individual vials with 2 ml viral transport medium (VTM). The components of the in-house prepared VTM included 25 g penicillin, 0.1 g ofloxacin, 0.2 g nystatin, 3.1 g polymycin B sulfate, 100 ml gentamycin, 2 g sulfamethoxazole, 0.4 g NaOH, and 19 g Medium 199 mixed with 2000 ml deionized water, which was then adjusted to pH 7.2. Viral enrichment was performed before sample extraction. In brief, 100 μl of supernatant was topped up to 1000 μl with PBS and filtered through a 0.22 μm pore-size Milex-GP filter to remove cells and debris. Two hundred μl filtrate was treated with 2 μl RNase A for 15 min at room temperature prior to treating with a mixture of nucleases (4 μl Turbo DNase, 30 μl 1 × Dnase buffer and 2 μl Benzonase) for 45 min at 37 °C to digest unprotected nucleic acids. Samples were immediately extracted using the NucliSENS® easyMag® system according to the manufacturer's instructions.

### Screening of astroviruses

RNA was screened for astroviruses using a pan-astrovirus heminested reverse transcription-PCR assay targeting the RdRp gene [22]. cDNA was generated from RNA using a PrimeScript™ RT reagent Kit in a 10 µl reaction. First-round PCR was performed using Ex Taq® DNA Polymerase Hot-Start Version. Nested PCR was performed using TaKaRa Taq™ DNA Polymerase Hot Start Version. A 25 µl reaction mixture was prepared according to the manufacturer's instructions. The thermocycling conditions for both PCR rounds were the same. Positive samples with a 422 bp amplicon were confirmed and identified through Sanger sequencing.

### Host identification

Positive samples were then subjected to previously described DNA barcoding for virus host identification [23]. This involved nested PCR using specific primers for avian mitochondrial DNA. Ex Taq® DNA Polymerase Hot-Start Version was used for the two-round PCR, according to the manufacturer's protocol. Confirmation of a 670 bp amplicon was done by Sanger sequencing and matching against the NCBI database for identification using BLAST.

### Phylogenetic and genomic analysis

The obtained sequences were aligned with astrovirus sequences downloaded from the NCBI database using MAFFT. The phylogenetic tree was built using IQ-TREE v1.6.12 with the GTR + I + G model and bootstrap 1000 times [24–26]. Eighteen samples were subjected to meta-transcriptome sequencing with the NovaSeq 6000 platform (Illumina, paired-end 150 bp) by Novogene (HK) Company Limited (Hong Kong, China). The workflow for constructing the meta-transcriptome sequencing library began with removing rRNAs. The remaining RNAs were then fragmented into approximately 250 to 300 bp fragments. These fragments were reverse-transcribed into double-stranded cDNAs using random primers, followed by end repair (150 bp), A-tailing, and adapter ligation. Subsequently, fragment size selection and PCR amplification were performed to prepare the library for sequencing. The raw data were processed to remove adapters and low-quality reads using fastp v 0.20.1 [27]. De novo assembly was performed using Trinity v2.1.1 [28]. The assembled contigs were examined employing Blastn and Blastx [29], referencing the entire non-redundant protein (nr) and nucleotide (nt) databases, in addition to the Astrovirus nt and nr databases acquired from NCBI (https://www.ncbi.nlm.nih.gov/), to identify astrovirus-associated contigs. The contigs were visualized and analyzed with Geneious Prime 2022.2.2 (https://www.geneious.com/). Genome polishing was performed using Pilon and BEDTools [30, 31]. Amino acid pairwise distances (p-dist) were calculated using MEGA 11 software [32]. ORF predictions were done using NCBI Open Reading Frame Finder (https://www.ncbi.nlm.nih.gov/orffinder/). Conserved domains were identified using NCBI Conserved Domain Search (https://www.ncbi.nlm.nih.gov/Structure/cdd/wrpsb.cgi). Transmembrane helices were predicted using TMHMM v2.0 (https://services.healthtech.dtu.dk/services/TMHMM-2.0/). The location of viral genome-linked protein (VPg) was predicted using FoldIndex© (https://fold.proteopedia.org/cgi-bin/findex). Nuclear localization signals (NLS) were identified using NLStradamus (http://www.moseslab.csb.utoronto.ca/NLStradamus/), and the coiled-coil region was checked using Multicoil Scoring Form https://cb.csail.mit.edu/cb/multicoil/cgi-bin/multicoil.cgi). Stem-loop II motifs (s2m) were predicted using the RNAfold web server (http://rna.tbi.univie.ac.at//cgi-bin/RNAWebSuite/RNAfold.cgi).

## Results

### Detection of astroviruses in bird fecal samples

A total of 1,712 bird fecal samples collected during the winters of 2018–2019, 2020–2021, and 2022–2023 in the Mai Po Marshlands were tested. Astrovirus-positive rates in the three periods were 8.5%, 12.8%, and 5.6%, respectively (Table 1). During 2022–2023, the highest positivity rate occurred in December. DNA barcoding was used to identify the host species, with 91.6% of the positive samples having the host species successfully identified (Table 2). The main species identified included *Mareca falcata*, *Anas acuta*, *Anas crecca*, and *Spatula clypeata* (Table 2). All identified bird species are consistent with the "Mai Po Bird Species List" in the World Wildlife Fund's 2022 report [33]. *Mareca falcata* is listed as near-threatened in global conservation status.

**Table 1:** Detection of astrovirus in wild bird samples in 2018–2019, 2020–2021, and 2022–2023 in Mai Po

Year / Month for sample collection	Total No. of samples	No. of AstV-positive samples (% of total)
2018–2019	94	8 (8.5)
2020–2021	94	12 (12.8)
2022–2023	1524	86 (5.6)
November 2022	216	18 (8.3)
December 2022	288	44 (15.3)
January 2023	288	3 (1.0)
February 2023	300	7 (2.3)
March 2023	288	10 (3.5)
April 2023	144	4 (2.8)
Total	1712	106

**Table 2:** Overview of host identification for AstV-Positive samples in 2018–2019, 2020–2021, and 2022–2023 in Mai Po

Avian (Order/Family)	Common Name (Species)	No. of AstV-positive samples (% of total)	No. of AstV-positive samples in different year		
			2018–2019	2020–2021	2022–2023
*Anseriformes/ Anatidae*	Falcated duck (*Mareca falcata*)	27 (25.5)	2	3	22
	Northern pintail (*Anas acuta*)	21 (19.8)	0	4	17
	Common teal (*Anas crecca*)	12 (11.3)	0	0	12
	Northern shoveler (*Spatula clypeata*)	11 (10.4)	2	2	7
	Eurasian wigeon (*Mareca penelope*)	5 (4.7)	0	0	5
	Mallard (*Anas platyrhynchos*)	1 (0.9)	0	1	0
*Charadriiformes/ Scolopacidae*	Redshank (*Tringa totanus*)	5 (4.7)	0	0	5
*Charadriiformes/Recurvirostridae*	Pied avocet (*Recurvirostra avosetta*)	3 (2.8)	0	0	3
*Charadriiformes/ Charadriidae*	Pacific golden plover (*Pluvialis fulva*)	3 (2.8)	0	0	3
	Lesser sand plover (*Charadrius mongolus*)	1 (0.9)	0	0	1
	Grey plover (*Pluvialis squatarola*)	1 (0.9)	0	0	1
*Pelecaniformes/ Arteidae*	Grey heron (*Ardea cinerea*)	4 (3.8)	1	0	3
*Suliformes/ Phalacrocoracidae*	Great cormorant (*Phalacrocorax carbo*)	3 (2.8)	1	2	0
Unidentified host		9 (8.5)	2	0	7
Total		106	8	12	86

### Phylogenetic analysis of partial astrovirus sequences

The partial RdRp sequences of our detected positive samples (*n* = 106) were used for comparison and phylogenetic analysis with previously known astrovirus sequences obtained from GenBank (*n* = 80, Table S1). Phylogenetic analysis revealed that most astroviruses (86 samples) detected in this experiment belonged to the genus *Avastrovirus*, 8 samples belonged to the genus *Mamastrovirus*, and 12 samples belonged to the unclassified astroviruses (related to aquatic host species) (Figure S1). Three recognized species, *Avastrovirus* 1–3, were identified following the ICTV 2019 classification and nomenclature. Newer unclassified viral clades are present in the phylogenetic analysis, such as *Avastrovirus* 4 and 5 (PasAstV), as previously described [34]. *Avastrovirus* 4 is a novel clade that includes samples we previously identified in 2009 and the samples from this study [21]. The classification will be further explained in the Discussion section.

Eighteen samples from genetically distinct clades, including *Avastrovirus* 4, and those from mamastrovirus and unclassified astroviruses, were selected for metagenomic analysis (Table S2). One sample, MP22-196 (PP623814, *Avastrovirus* 4), returned a near-complete genome sequence, while partial sequences of unclassified astrovirus were found in two other samples, MP18-799 and MP22-114 (Table 3). For the remaining 15 samples, despite testing positive in the heminested RT-PCR screening assay, they each had a poor RNA yield after the initial nucleic acids extraction. No astrovirus-like sequence was detected in these 15 studied samples.

**Table 3:** Astrovirus sequences detected in MP18-799 and MP22-114

Clades	Sample name ​	Contig Length (bp)​	Read	Result of BlastX		
				Viruses	Accession No	Region nt (ORFs)
*Avastrovirus* 4	MP18-799​	371​	52	Bastrovirus-like virus	NC_032426	374–744 (ORF1a​)
Unclassified astroviruses	MP22-114​	471	44	Blencathra virus	MT993595	4073–4547 with 3 gaps (ORF1b​)
		201	23	Blencathra virus	MT993595	4563–4812 with 49 gaps (ORF1b​)
		150	20	Blencathra virus	MT993595	4813–4964 with 2 gaps (ORF1b​)

### Genome characterization of Avastrovirus 4

**Fig. 1:**
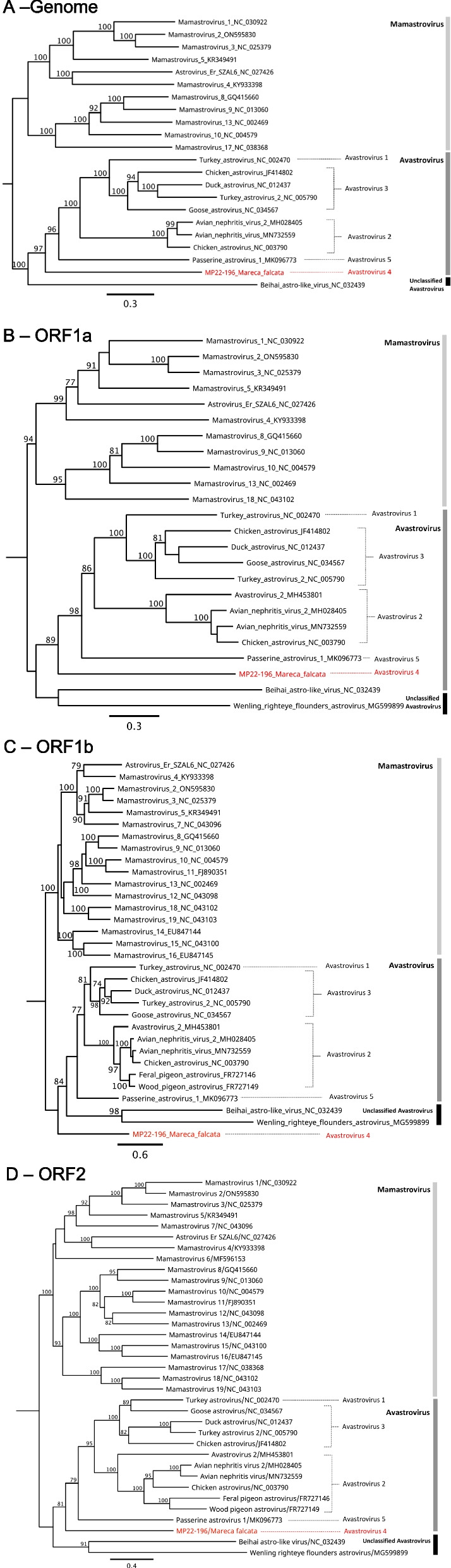
Phylogenetic analysis of MP22-196 and representative astrovirus strains of each clade based on **A** full genome sequences, **B** ORF1a, **C** ORF1b, and **D** ORF2 region using IQ‐TREE by maximum likelihood. The trees are rooted by the *Mamastrovirus* clade. The branch values are bootstrap supports (%) with 1000 replicates as statistical support. MP22-196 detected from this study are highlighted in red color. Representative astrovirus references (*n* = 41) of different clades with GenBank accession numbers are shown. According to the ICTV taxonomic classification, *Mamastrovirus* 1 to 19 are selected as references in the *Mamastrovirus* clade. The *Avastrovirus* strains selected include one reference from *Avastrovirus* 1, six references from *Avastrovirus* 2, four references from *Avastrovirus* 3, and one reference from *Avastrovirus* 5. Two representative unclassified Astrovirus references are also selected. It is worth noting that some references have only partial sequences so they are not included in some phylogenetic trees

We further extracted reference sequences (*n* = 41) to undergo phylogenetic analysis including *Mamastrovirus* 1–19 and *Avastrovirus* 1–5, along with MP22-196 (Fig. 1). The phylogenetic results of the full genome (Fig. 1A), ORF1a (Fig. 1B), or ORF2 (Fig. 1D) confirmed that MP22-196 is a new genetic lineage closely related to *Avastrovirus* and distinct from *Mamastrovirus*. Interestingly, in the ORF1b phylogenetic tree (Fig. 1C), MP22-196 appears as an independent branch outside the *Avastrovirus* genus. The current astrovirus classification of ICTV is based on the host and p-distance of the capsid region [35]. Pairwise comparisons between MP22-196 and these reference sequences were performed to calculate nucleotide (nt) and amino acid (aa) identity and p-distance (Table 4). For ORF1b, MP22-196 showed the highest nt identity (67.72–93.08%) and aa identity (66.14–98.46%) with partial clade 4 avastrovirus sequences previously detected in Hong Kong and Sweden. By contrast, the nt identity and aa identity of MP22-196 with avastroviruses from other clades are much lower (< 45%). The p-distance results between MP22-196 and each *Avastrovirus* 1–5 clades (0.697 to 0.722) were met with ICTV classification criteria, which requires the average p-distances between sub-clades to be within 0.576 to 0.742. However, MP22-196 had higher p-distances with *Mamastrovirus* and unclassified astroviruses, indicating its distance from these reference sequences.

**Table 4:** Estimates of percentage nucleotide and amino acid identity and genetic distances between MP22-196 and references

			MP22-196				
			Percentage identity (%)				p-distance
Clade	Viruses	Accession No	Nt	ORF1a (aa)	ORF1b (aa)	ORF2 (aa)	ORF2 (aa)
*Mamastrovirus*	Mamastrovirus 1	NC_030922	29.43	12.58	NA^a^	11.65	0.804
	Mamastrovirus 2	ON595830	29.17	11.51	36.58	14.43	0.762
	Mamastrovirus 3	NC_025379	29.31	11.99	34.78	15.39	0.744
	Mamastrovirus 4	KY933398	28.42	11.40	36.07	13.92	0.757
	Mamastrovirus 5	KR349491	25.50	NA	41.58	14.54	0.764
	Mamastrovirus 6	MF596153	20.40	NA	NA	13.62	0.796
	Mamastrovirus 7	NC_043096	32.88	NA	35.8	14.42	0.759
	Mamastrovirus 8	GQ415660	29.45	11.67	37.69	15.89	0.724
	Mamastrovirus 9	NC_013060	30.44	12.82	38.52	15.73	0.734
	Mamastrovirus 10	NC_004579	29.13	13.08	36.19	15.69	0.734
	Mamastrovirus 11	FJ890351	31.30	NA	33.93	16.69	0.732
	Mamastrovirus 12	NC_043098	23.88	NA	37.36	17.25	0.722
	Mamastrovirus 13	NC_002469	29.72	12.36	35.48	16.48	0.719
	Mamastrovirus 14	EU847144	30.25	NA	38.98	14.60	0.759
	Mamastrovirus 15	NC_043100	34.77	NA	39.59	16.37	0.729
	Mamastrovirus 16	EU847145	32.49	NA	35.97	16.29	0.741
	Mamastrovirus 17	NC_038368	23.65	NA	NA	14.99	0.737
	Mamastrovirus 18	NC_043102	29.76	12.56	35.61	14.16	0.742
	Mamastrovirus 19	NC_043103	26.28	NA	36.95	14.19	0.737
	Astrovirus Er SZAL6	NC_027426	28.19	12.44	35.76	13.50	0.771
*Avastrovirus* 1	Turkey astrovirus	NC_002470	34.26	17.96	41.01	20.47	0.702
*Avastrovirus* 2	Avastrovirus 2	MH453801	32.88	16.93	41.02	20.81	0.697^b^
	Avian nephritis virus 2	MH028405	31.14	17.29	41.25	19.53	0.722
	Avian nephritis virus	MN732559	31.85	17.48	41.05	19.78	0.712
	Chicken astrovirus	NC_003790	31.65	17.22	40.63	**20.87**^c^	0.714
	Feral pigeon astrovirus	FR727146	32.65	NA	43.45	18.47	0.712
	Wood pigeon astrovirus	FR727149	32.09	NA	44.20	19.73	0.699
*Avastrovirus* 3	Chicken astrovirus	JF414802	31.65	17.22	38.92	**20.87**	0.714
	Duck astrovirus	NC_012437	32.08	16.47	37.84	18.37	0.707
	Goose astrovirus	NC_034567	33.32	17.57	39.73	19.12	0.714
	Turkey astrovirus 2	NC_005790	33.16	**18.12**	38.83	17.81	0.704
*Avastrovirus* 4 ^d^	Avastrovirus 3 MPJ0552	JX985682	67.72	NA	66.14	NA	NA
	Avastrovirus 3 MPJ1332	JX985700	76.34	NA	80.65	NA	NA
	Avastrovirus 3 MPJ1348	JX985704	77.6	NA	82.03	NA	NA
	Avastrovirus 3 MPJ1364	JX985709	**93.08**	NA	**98.46**	NA	NA
	Avastrovirus 3 MPJ1433	JX985714	72.85	NA	70.97	NA	NA
	Avastrovirus 3 MPJ1442	JX985715	73.12	NA	70.97	NA	NA
	Avastrovirus 3 Sweden 701	KY320411	75	NA	82.4	NA	NA
*Avastrovirus* 5	Passerine astrovirus 1	MK096773	30.58	15.01	36.86	19.26	0.709
Unclassified astroviruses	Beihai astro-like virus	NC_032439	25.34	10.97	34.74	11.17	0.836
	Wenling righteye flounders astrovirus	MG599899	24.20	11.24	28.06	11.76	0.765

^a^No sequences available of specific ORF region in the public database

^b^Underlined values are the lowest and highest p-distance

^c^Bolded value is the highest percentage identity (%) of each ORF region

^d^The reference sequence identified as *Avastrovirus* 4 only has RdRp region (ORF1b)

**Fig. 2:**
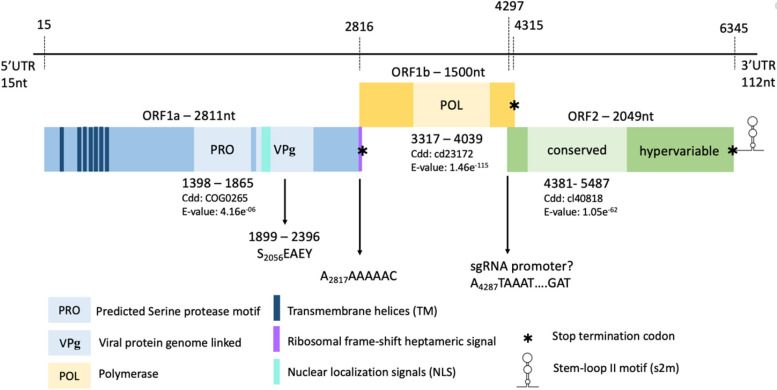
Schematic representation of the near-complete genome organization of MP22-196. The line at the upper indicated the nucleotide (nt) length and the dashed line stated the location of 5' UTR, 3' UTR, and the starting and final nucleotide for ORF. The total nucleotide length (upper rectangles), different conserved domain hits with nucleotide location, accession number (Cdd), and E-value (below rectangles) are shown in each ORF. TEEEY-like motif and ribosomal frame-shift heptameric signal are stated at ORF1a. Putative sgRNA promoter is indicated between the end of ORF1b and the start of ORF2

**Fig. 3:**
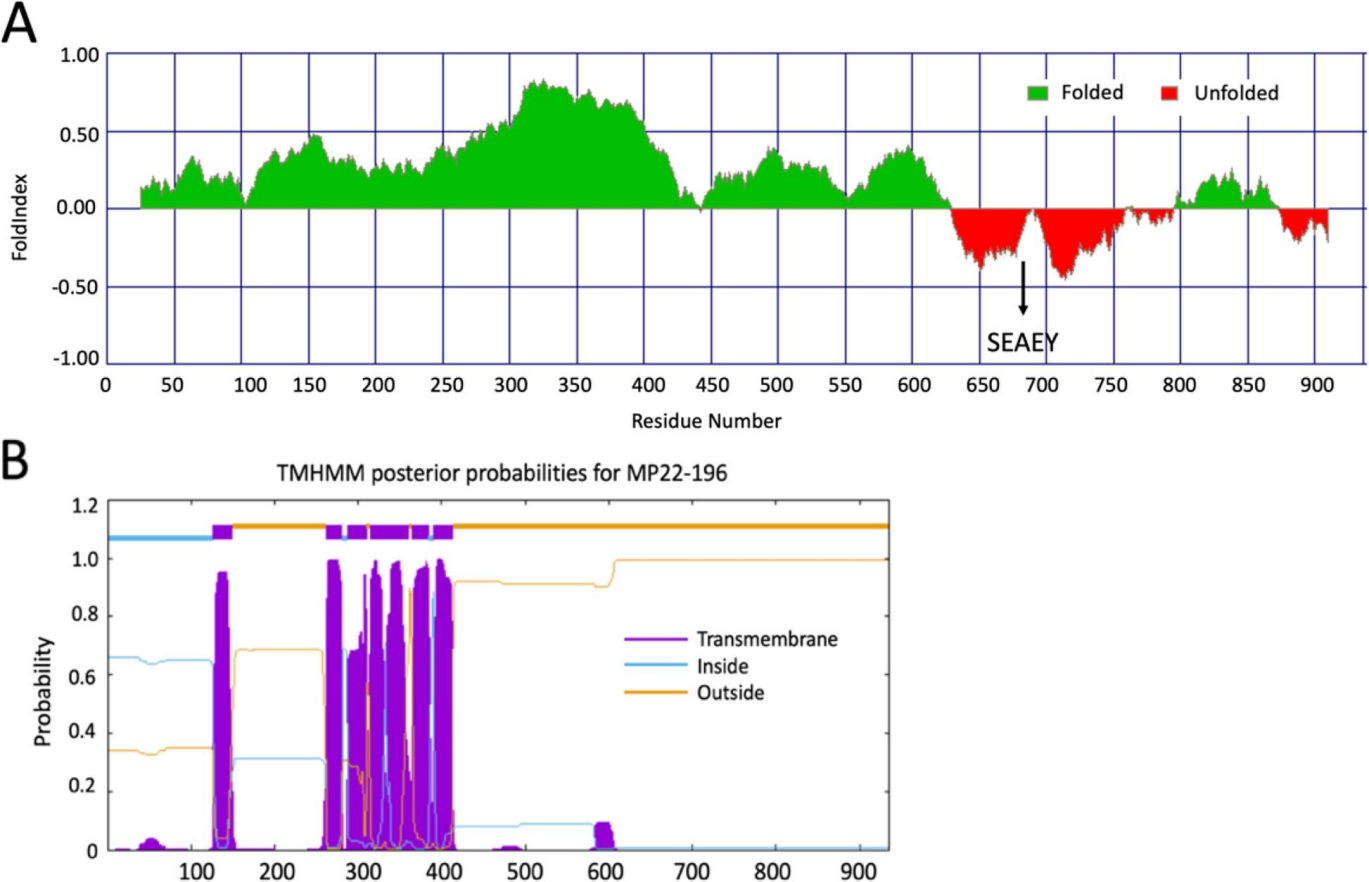
**A** Prediction of putative viral protein associated with the genome (VPg) domain by FoldIndex and indicated TEEEY-like motif (at 681 to 685). **B** Prediction of transmembrane helices in proteins by TMHMM and stated as the peak with purple color

**Fig. 4:**
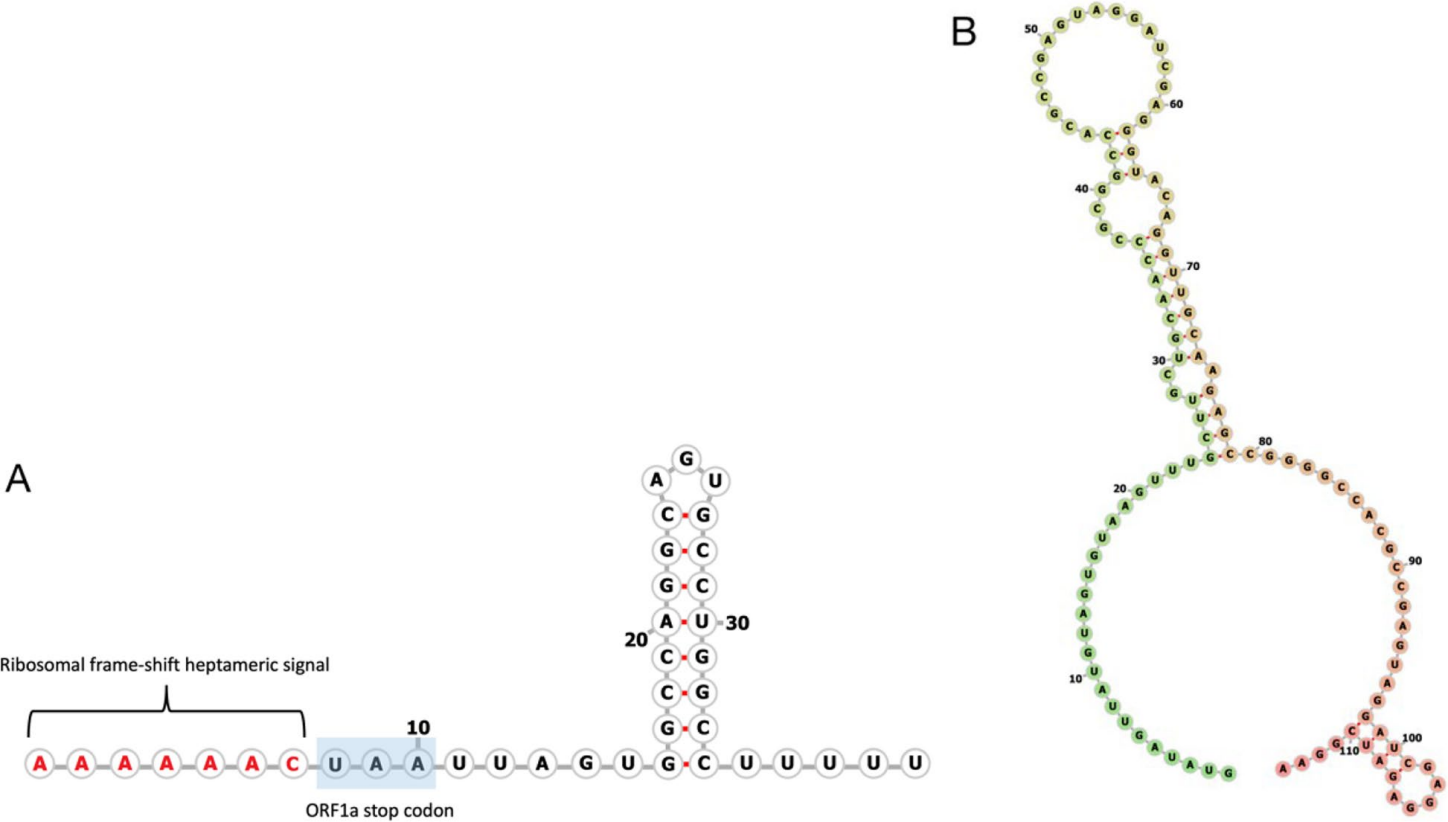
**A** The predicted secondary structure of ribosomal frame-shift at the ORF1a 3′end. The stop codon of the ORF1a is shown represented by a blue rectangle. **B** The predicted secondary structure of stem-loop 2 motifs (s2m) at 3′UTR

**Fig. 5:**
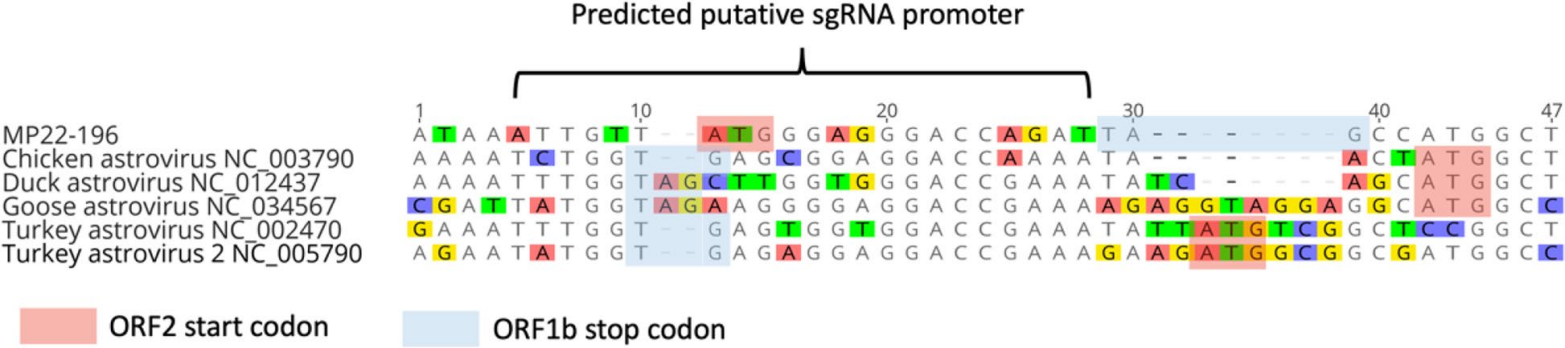
Alignment of the partial nucleotide sequences between the ORF1b and ORF2 of MP22-196 and other *Avastrovirus* strains. Prediction of putative sgRNA promoter indicated in the figure. The sequence variation is highlighted in color. The start codon of the ORF2 and the stop codon of the ORF1b are represented by red and blue rectangles, respectively

The deduced near-complete genome of MP22-196 is 6566 nt long, with a mean sequence coverage of 2,469 times. The viral genome includes 5' and 3' untranslated regions (UTR) and consists of three open reading frames (ORF), namely ORF1a (2811 nt), ORF1b (1500 nt), and ORF2 (2049 nt) (Fig. 2). ORF1a encodes non-structural proteins (NSP) and includes transmembrane domains (TM) (Fig. 3B), a serine protease (PRO), a VPg region (Fig. 3A), an NLS, and a heptanucleotide ribosomal frameshift signal (A_2817_AAAAAC) (Fig. 4A). A putative monopartite NLS (K_1974_KKGKTKKGRGSRINAVRKALRRMK_2048_) was discovered. Although the conserved TEEEY amino acid motif was not recognized, the VPg region has a TEEEY-like residue (S_2096_EAEY). The common ribosomal frameshift heptamer signal of astrovirus was found at the 3' end of ORF1a, followed by the stop codon of ORF1a and stem-loop structure (Fig. 4A). ORF1b overlaps with ORF1a and contains the RNA polymerase coding motif (3317 to 4039 nt). Its expression is mediated through -1 ribosome frameshift. When the programmed ribosomal frameshift mechanism occurs, the non-structural astrovirus protein is translated into two polyproteins, nsp1a (104 kDa) and nsp1ab (159 kDa). The VPg region of ORF1a and the RdRp motif in ORF1b are responsible for replication and virus particle production [36]. ORF2 encodes structural proteins, including the capsid protein precursor, with conserved (4381 to 5487 nt) and variable domains. The conserved domain is responsible for astrovirus capsidization and virus particle formation [37, 38]. The variable domain is related to virus tropism, neutralizing epitopes, and serotype differentiation [12]. The conserved astrovirus promoter sequence motif 5'-AUUUGGANGNGGNGGACCNAAN_(1–9)_AUG-3' of the viral subgenomic RNA (sgRNA) could not be accurately identified. However, a potential but modified sgRNA sequence was found near the ORF2 start codon. It can be aligned with other sequenced avian astroviruses. Interestingly, the ORF2 start codon is placed before the ORF1b stop codon, which differs from other avastrovirus sequences (Fig. 5). The deduced 5' and 3' UTR of MP22-196 are 15 nt and 160 nt long, respectively, with a poly(A) tail. The 3' UTR contains a highly conserved s2m sequence (5'-CCCGCGGCCACCGCCGAGTAGGATCGAGGGTACAG-'3) in the *Astroviridae* family (Fig. 4B) [39].

## Discussion

We previously detected novel astroviruses in wild birds in 2010–2011 [21]. The current study applied a similar strategy to screen for astroviruses in wild bird fecal samples collected from 2018–2023 at the same site. The positive rate for DNA barcoding assay for host identification in the current study fluctuated between 57 and 93% [23]. The results of this experiment have a high positive rate of 91.6%, which is similar to the results of others [40, 41]. This allowed us to observe the evolution of astroviruses in wild birds over the past decade and gain insight into their complete genome sequencing through metagenomics.

During the winter months (November to April), our surveillance team collected samples from shallow waters and mudflats in Mai Po. The positive rate for astrovirus detection varied throughout the months, with December having the highest rate (15.3%) and the other months showing lower rates (8.3% to 1%) (Table 1). Compared to our 2010 survey, avian diversity with positive astrovirus detection is higher during 2022–2023, indicating interspecies transmission between populations and a high risk of virus transmission among different wild birds [21].

We performed metagenomic sequencing on 18 samples to understand viral communities and their functional characteristics [42]. A near-complete astrovirus genome from the *Avastrovirus* 4 clade was successfully obtained in Sample MP22-196. To understand the characteristics of the *Avastrovirus* 4 clade, we used the nearly complete genome and different ORF regions to conduct phylogenetic analyses (Fig. 1). The results of these phylogenetic trees were similar. Only the ORF1b region phylogenetic tree suggests MP22-196 is an outlier of *Avastroviruses*. To further confirm that MP22-196 belongs to the *Avastrovirus* 4 branch and its relationship with each clade, we calculated the aa identity of each ORF region with the representative reference sequence and the p-distance of its ORF2. The highest aa identity (66.14–98.46%) was presented at ORF1b of MP22-196 with *Avastrovirus* 4 reference sequences which prove it to be closely related to this clade. Although ORF1b phylogenetic result suggests MP22-196 is genetically distinct from other avastroviruses, it shares a relatively higher aa identity with *Avastrovirus* 1 (41.01%) and *Avastrovirus* 2 (40.63–44.20%). According to the ICTV classification criteria, the average p-distance between clades should be 0.576 to 0.742, while that within a clade should be 0.204 to 0.284 [35]. Average p-distances of MP22-196 with each *Avastrovirus* clade (0.702–0.710) meet the criteria for distinguishing between clades. The result also showed our sample distance from *Mamastrovirus* or unclassified astroviruses. This supports that MP22-196 is closely related to the *Avastrovirus* genus. Whole-gene sequencing of *Avastrovirus* 4 has not been reported, and our results can provide valuable insights into its characteristics, evolution, and transmission by wild waterfowl.

Mamastroviruses detected in avian hosts are rare. While a prior study identified mammalian-like astroviruses in wild birds [18], our research further substantiates the presence of mamastroviruses in avian hosts through phylogenetic and barcoding analyses. The eight mamastroviruses we examined exhibit close relationships with porcine and dromedary astroviruses; however, no relevant host genes from the *Suidae* and *Camelidae* families were discovered in the metagenomic data. Our bar-coding analysis also confirmed that these fecal samples were of avian origin (Figure S1). Our detection of mamastroviruses in avian hosts raises potential concerns regarding cross-species transmission. However, other hypotheses, such as environmental contamination or ingestion of food containing mamastroviruses by these birds, cannot be excluded. Further investigation on this topic is warranted.

Our phylogenetic analysis identified 5 distinct clades of *Avastrovirus* (Figure S1). *Avastrovirus* 1, 2, and 3 are closely related, followed by *Avastrovirus* 5. *Avastrovirus* 3 was the most prevalent in our wild waterfowl samples, accounting for 58.5% of the positive samples. According to the current ICTV virus classification, *Avastrovirus* 3 primarily infects ducks and turkeys, with reference gene sequences being Duck astrovirus (NC_012437) and Turkey astrovirus 2 (NC_005790). Notably, *Avastrovirus* 4 detected by us is genetically distinct from *Avastrovirus* 3. In a previous investigation in 2010, we grouped *Avastrovirus* 4 as *Avastrovirus* 3 based on limited pre-existing astrovirus sequences. But our latest phylogenetic analyses indicate that they are more distantly related. Therefore, it is more appropriate to differentiate them into 2 different genetic clades. Based on partial sequences, Fernadez-Correa et al*.* also referred to this clade as *Avastrovirus* 4 [34]. *Avastrovirus* 4 exhibits a high degree of host specificity, as observed in a 2010 survey [21]. The main hosts of this clade are from the Order of *Anseriformes* and the Family of *Anatidae*. The species include *Anas acuta*, *Anas crecca*, *Mareca falcata,* and *Spatula clypeata*. There is only one specimen from the *Ardea cinerea*, which belongs to the Order of *Pelecaniformes* and Family of *Arteidae*.

The virus genome of MP22-196 has three open reading frames: ORF1a and ORF1b, which encode non-structural proteins involved in viral transcription and replication, and ORF2, which encodes the capsid polyprotein necessary for viral assembly [12, 36]. Several key features were observed (Fig. 2). First, the ORF1a stop codon is located after the highly conserved heptamer signal and before the stem-loop structure, a typical characteristic of avian astroviruses (Fig. 4A) [43, 44]. Ribosomal frameshifting is induced by the above two cis-acting elements [45, 46]. Normally, when translating ORF1a, the production of nsp1a stops at the stop codon. When the ribosomal frameshift mechanism occurs, the -1 frameshift at the overlap of ORF1a and ORF1b will allow the viral polymerase to translate into nsp1ab [45]. However, a previous study indicated that this distinctive feature affects the ability to induce frameshift in avian and suggests they may use different strategies for translating ORF1ab compared to human astroviruses [2]. The number of resulting non-structural proteins is not yet fully elucidated. Further studies are needed to elucidate the translation mechanisms and encoded non-structural proteins of this *Avastrovirus* 4 clade. Second, the putative sgRNA sequence differs from other avian astrovirus in that the ORF2 start codon is positioned before ORF1b. The impact of this start codon positioning on the sgRNA sequence remains to be determined. Previous research has indicated that human astroviruses frequently display a slight overlap between the ORF1b and ORF2 regions, whereas it is rare in avian astroviruses [47, 48]. Third, the 3' UTR of MP22-196 is shorter (160 nt) than other avian astroviruses [2]. The impact of 3' UTR shortening on avian astroviruses remains unknown. However, a study in human astrovirus pointed out that 3' UTR deletion eliminates viral protein expression [49]. Also, shortened 3' UTR has fewer protein binding sites in the porcine astrovirus 3 [50]. We didn't conduct 5' and 3' RACE to ascertain the ends of the viral genome. Through alignment with other avastrovirus genomes, we estimate that our deduced viral sequence misses the first 6 bases of viral RNA. Deduction of the 5' end of the MP22-196 would require further experimental work (e.g. 5' RACE). In contrast, the deduced 3' end ended with a short poly(A) tail, indicating that it is an authentic 3' end sequence. Fourth, a highly conserved s2m element was found in the 3' UTR, which some literature indicates that it may affect viral and host cellular proteins in RNA replication, though its exact function is not fully understood [51].

This study reveals the first complete sequence of *Avastrovirus* 4, along with its phylogenetic analysis and genome organization. *Avastrovirus* 4 has been detected in wild winter migratory birds in Hong Kong since 2009. These birds likely act as reservoirs. Ongoing surveillance of avian astroviruses in Hong Kong's wild birds can provide insights into their evolution, geographical distribution, and host relationships.

### Supplementary Information

Supplementary Material 1.

## Data Availability

No datasets were generated or analysed during the current study.
