# Supplementary material for: Genetic diversity of astroviruses detected in wild aquatic birds in Hong Kong
Source: Virol J. 2024 Jul 7;21:153. doi: 10.1186/s12985-024-02423-w (PMC11229208; doi:10.1186/s12985-024-02423-w)
Supplement: Supplementary file 1 — Supplementary Material 1. [file 12985_2024_2423_MOESM1_ESM.docx]

| **Table S1.** Sequences used for phylogenetic analysis as shown in Figure S1. Reference sequences (n=80) in different clade of astrovirus were downloaded from Genbank. | | | | | | | | | | |
| --- | --- | --- | --- | --- | --- | --- | --- | --- | --- | --- |
|  |  |  |  | **Length** | | |  |  |  |  |
| **Clades** | **Sequence Name** | **Accession No.** | **Total Length** | **ORF1a** | **ORF1b** | **ORF2** | **Collection Date** | **Country** | **Host** | **Strain** |
| Mamastrovirus | Astrovirus Er/SZAL6/European roller/Hungary/2011/NC_027426 | NC_027426 | 7025 | 2826 | 1485 | 2772 | 2011-07 | Hungary | Coracias garrulus | Er/SZAL6/HUN/2011 |
| Mamastrovirus | Astrovirus MLB1/Australia/1999/NC_011400 | NC_011400 | 6171 | 2364 | 1536 | 2271 | 1999-10-06 | Australia | - | MLB1 |
| Mamastrovirus | Astrovirus MLB2/Human/USA/2008/NC_016155 | NC_016155 | 6119 | 2364 | 1536 | 2238 | 2008 | USA | Homo sapiens | MLB2/human/Stl/WD0559/2008 |
| Mamastrovirus | Astrovirus MLB3/Human/India/2004/NC_019028 | NC_019028 | 6124 | 2364 | 1536 | 2244 | 2004 | India | Homo sapiens | MLB3/human/Vellore/26564/2004 |
| Mamastrovirus | Astrovirus VA3/India/2005/NC_019026 | NC_019026 | 6581 | 2670 | 1662 | 2268 | 2005 | India | Homo sapiens | VA3/human/Vellore/28054/2005 |
| Mamastrovirus | Astrovirus VA4/Human/Nepal/2008/NC_019027 | NC_019027 | 6518 | 2661 | 1581 | 2190 | 2008 | Nepal | Homo sapiens | VA4/human/Nepal/S5363/2008 |
| Mamastrovirus | Bovine astrovirus/Hong Kong/NC_023632 | NC_023632 | 6317 | 2403 | 1509 | 2358 | 2011 | China: Hong Kong | Cattle | B170/HK |
| Mamastrovirus | Bovine astrovirus/Switzerland/2012/NC_024498 | NC_024498 | 6530 | 2534 | 1576 | 2286 | 2012-03 | Switzerland | Cattle | CH13 |
| Mamastrovirus | California sea lion astrovirus 2/USA/2008/NC_034975 | NC_034975 | 3510 | - | 987 | 2580 | 2008 | USA | Zalophus californianus | CSL2 |
| Mamastrovirus | Canine astrovirus strain Gillingham/UK/2012/NC_026814 | NC_026814 | 6617 | 2670 | 1536 | 2505 | 2012-08 | United Kingdom | Canine | Gillingham/2012/UK |
| Mamastrovirus | Deer astrovirus/Denmark/2010/HM447045 | HM447045 | 3051 | - | 717 | 2268 | 2010 | Denmark | Capreolus capreolus | VS3600005 |
| Mamastrovirus | Dromedary astrovirus/United Arab Emirates/2013/NC_027711 | NC_027711 | 6327 | 2475 | 1511 | 2262 | 2013-04-04 | United Arab Emirates | Camelus dromedarius | DcAstV-274 |
| Mamastrovirus | Feline astrovirus 2/Hong Kong/2012/NC_022249 | NC_022249 | 6795 | 2745 | 1545 | 2445 | 2012-03-01 | China: Hong Kong | Felis catus | 1637F |
| Mamastrovirus | Feline astrovirus D1/USA/2013/NC_024701 | NC_024701 | 6598 | 2547 | 1539 | 2355 | 2013-10 | USA | Felis catus | FAstV-D1 |
| Mamastrovirus | HMO astrovirus A/Nigeria/2007/NC_013443 | NC_013443 | 6534 | 2615 | 1573 | 2196 | 2007-03-21 | Nigeria | Homo sapiens | NI-295 |
| Mamastrovirus | Human astrovirus BF34/2010/NC_024472 | NC_024472 | 6577 | 2667 | 1570 | 2406 | 2010 | Burkina Faso | Homo sapiens | BF34 |
| Mamastrovirus | Human astrovirus/NC_001943 | NC_001943 | 6813 | 2760 | 1536 | 2361 | 1994 | - | Homo sapiens | - |
| Mamastrovirus | Mamastrovirus 1 Human astrovirus/India/2004/NC_030922 | NC_030922 | 6673 | 4296 (1ab) | | 2385 | 2004-09-02 | India | Homo sapiens | V1347 |
| Mamastrovirus | Mamastrovirus 2 Feline astrovirus/Australia/2016/ON595830 | ON595830 | 6833 | 2745 | 1473 | 2451 | 2016 | Australia | Felis catus | FPV-8 |
| Mamastrovirus | Mamastrovirus 3 Porcine astrovirus/China/2013/NC_025379 | NC_025379 | 6705 | 2721 | 1553 | 2340 | 2013 | China | Swine | PAstV-GX1 |
| Mamastrovirus | Mamastrovirus 4 Porcine astrovirus/Kenya/2012/KY933398 | KY933398 | 6649 | 2487 | 1509 | 2511 | 2012-08 | Kenya | Swine | K456 |
| Mamastrovirus | Mamastrovirus 5 Dog astrovirus/Brazil/2013/KR349491 | KR349491 | 4981 | 1167 | 609 | 2502 | 2013 | Brazil | Canis lupus familiaris | MAstV5_Grav/2013/BRA |
| Mamastrovirus | Mamastrovirus 6 Human astrovirus FSS656/Australia/2015/MF596153 | MF596153 | 1977 | - | - | 1977 | 2015-12-28 | Australia | Homo sapiens | Qld-FSS656 |
| Mamastrovirus | Mamastrovirus 7 Bottlenose dolphin astrovirus 1/USA/2007/NC_043096 | NC_043096 | 3990 | - | 1420 | 2481 | 2007 | USA | Tursiops truncatus | Bd1 |
| Mamastrovirus | Mamastrovirus 8 Human astrovirus NI-295/Nigeria/2007/GQ415660 | GQ415660 | 6534 | 2615 | 1573 | 2196 | 2007-03-21 | Nigeria | Homo sapiens | NI-295 |
| Mamastrovirus | Mamastrovirus 9 Human astrovirus VA1/USA/2008/NC_013060 | NC_013060 | 6586 | 2661 | 1569 | 2277 | 2008-08 | USA | Homo sapiens | VA1 |
| Mamastrovirus | Mamastrovirus 10 Mink astrovirus/NC_004579 | NC_004579 | 6610 | 2625 | 1557 | 2328 | 2003 | - | Mink | - |
| Mamastrovirus | Mamastrovirus 11 California sea lion astrovirus/USA/2006/FJ890351 | FJ890351 | 3179 | - | 829 | 2343 | 2006 | USA | Zalophus californianus | CSL1 |
| Mamastrovirus | Mamastrovirus 12 Bat astrovirus/China/2007/NC_043098 | NC_043098 | 3975 | - | 1641 | 2283 | 2007-06-08 | China | Taphozous melanopogon | LD71 |
| Mamastrovirus | Mamastrovirus 13 Ovine astrovirus/NC_002469 | NC_002469 | 6440 | 2535 | 1566 | 2289 | 2003 | Scotland | Ovine | - |
| Mamastrovirus | Mamastrovirus 14 Bat astrovirus/Hong Kong/2005/EU847144 | EU847144 | 3371 | - | 753 | 2217 | 2005 | China: Hong Kong | Miniopterus magnater | AFCD57 |
| Mamastrovirus | Mamastrovirus 15 Bat astrovirus/China/2007/NC_043100 | NC_043100 | 3875 | - | 1413 | 2163 | 2007-06-08 | China | Taphozous melanopogon | LD77 |
| Mamastrovirus | Mamastrovirus 16 Bat astrovirus/Hong Kong/2005/EU847145 | EU847145 | 3180 | - | 825 | 2124 | 2005 | China: Hong Kong | Pipistrellus abramus | AFCD11 |
| Mamastrovirus | Mamastrovirus 17 Bat astrovirus Hp LC03/China Guangxi/2007/NC_038368 | NC_038368 | 2403 | - | - | 2403 | 2007-06-06 | China | Hipposideros pomona | LC03 |
| Mamastrovirus | Mamastrovirus 18 Bat astrovirus/Hong Kong/2006/NC_043102 | NC_043102 | 5083 | 909 | 1572 | 2553 | 2006 | China: Hong Kong | Miniopterus pusillus | AFCD337 |
| Mamastrovirus | Mamastrovirus 19 Bat astrovirus/China/2007/NC_043103 | NC_043103 | 4241 | - | 1569 | 2538 | 2007-06-08 | China | Taphozous melanopogon | LD38 |
| Mamastrovirus | Minke whale astrovirus 2/USA/2003/HQ668143 | HQ668143 | 3099 | - | 730 | 2340 | 2003 | USA | Balaenoptera acutorostrata | MH03-629 |
| Mamastrovirus | Mouse astrovirus/USA/2008/NC_015935 | NC_015935 | 6543 | 2550 | - | 2124 | 2008 | USA | Mus musculus | M-52 |
| Mamastrovirus | Murine astrovirus/USA/2011/NC_018702 | NC_018702 | 6838 | 2784 | 1506 | 2457 | 2011-01 | USA | Murine | STL 1 |
| Mamastrovirus | Wild boar astrovirus/Hungary/2011/NC_016896 | NC_016896 | 6707 | 2559 | 1569 | 2538 | 2011-04 | Hungary | Sus scrofa | wild boar/WBAstV-1/2011/HUN |
| Mamastrovirus | Porcine astrovirus 2/USA/2010/NC_023674 | NC_023674 | 6318 | 2475 | 1511 | 2250 | 2010 | USA | Sus scrofa | 43/USA |
| Mamastrovirus | Porcine astrovirus 3/USA/2011/NC_019494 | NC_019494 | 6460 | 2535 | 1570 | 2454 | 2011-06-24 | USA | Sus scrofa | US-MO123 |
| Mamastrovirus | Porcine astrovirus 4/USA/2010/NC_023675 | NC_023675 | 6639 | 2553 | 1517 | 2478 | 2010 | USA | Sus scrofa | 35/USA |
| Mamastrovirus | Porcine astrovirus 5/USA/2011/NC_023636 | NC_023636 | 6500 | 2619 | 1511 | 2346 | 2011-06-23 | USA | Sus scrofa | AstV5-US-IA122 |
| Mamastrovirus | Qinghai Himalayan marmot astrovirus 1/China/2013/NC_033792 | NC_033792 | 6712 | 2664 | 1539 | 2418 | 2013/2014 | China | Marmota himalayana | HHMAstV1 |
| Mamastrovirus | Qinghai Himalayan marmot astrovirus 2/China/2013/NC_033821 | NC_033821 | 6640 | 2661 | 1533 | 2367 | 2013/2014 | China | Marmota himalayana | HHMAstV2 |
| Mamastrovirus | Rabbit astrovirus/USA/2010/NC_025346 | NC_025346 | 7353 | 3204 | 1533 | 2559 | 2010-06-04 | USA | Oryctolagus cuniculus | TN rabbit 10-2208 |
| Mamastrovirus | Rat astrovirus/Hong Kong/2007/HM450381 | HM450381 | 4940 | 941 | 1551 | 2442 | 2007 | China: Hong Kong | Rattus norvegicus | RS118 |
| Mamastrovirus | Rodent astrovirus/China/NC_036583 | NC_036583 | 6861 | 2742 | 1515 | 2586 | 2017 | China | Rattus norvegicus | GX-006 |
| Mamastrovirus | Sichuan takin astrovirus/China/2013/NC_037655 | NC_037655 | 6233 | 2307 | 1509 | 2451 | 2013 | China | Budorcas taxicolor tibetana | LLT03 |
| Avastrovirus 1 | Turkey astrovirus/NC_002470 | NC_002470 | 7003 | 3300 | 1534 | 2016 | 2000 | USA | Turkey | - |
| Avastrovirus 2 | Avastrovirus 2 isolate MPJ0829/Platalea minor/Hong Kong/2009/JX985689 | JX985689 | 360 | - | 360 | - | 2009-12-17 | China: Hong Kong | Platalea minor | MPJ0829 |
| Avastrovirus 2 | Avastrovirus 2 isolate MPJ0918/Tringa nebularia/Hong Kong/2009/JX985690 | JX985690 | 399 | - | 399 | - | 2009-12-17 | China: Hong Kong | Tringa nebularia | MPJ0918 |
| Avastrovirus 2 | Avastrovirus 2/Red-necked Avocet/Australia/2013/MH453801 | MH453801 | 6869 | 2992 | 1529 | 2046 | 2013 | Australia | Red-necked Avocet | AstV/Red-necked Avocet/MW09/Interior |
| Avastrovirus 2 | Avian nephritis virus 2/Brazil/2009/MH028405 | MH028405 | 6923 | 4584 (1ab) | | 1944 | 2009 | Brazil | Gallus gallus | AVE52/ANV2 |
| Avastrovirus 2 | Avian nephritis virus/Gallus gallus/China/2017/MN732559 | MN732559 | 6885 | 3017 | 1528 | 2004 | 2017 | China | Gallus gallus | ANV/CHN/GXJL815/2017 |
| Avastrovirus 2 | Chicken astrovirus/NC_003790 | NC_003790 | 6927 | 3015 | 1524 | 2052 | 2000 | - | Chicken | G-4260 |
| Avastrovirus 2 | Feral pigeon astrovirus/Norway/2003/FR727146 | FR727146 | 3263 | - | 942 | 2025 | 2003-08-19 | Norway | Feral pigeon | 03/594-6 |
| Avastrovirus 2 | Wood pigeon astrovirus/Norway/2005/FR727149 | FR727149 | 3307 | - | 960 | 2031 | 2005-08-23 | Norway | Wood pigeon | 06/15660-1 |
| Avastrovirus 3 | Avastrovirus 1 isolate MPJ0597/Anas clypeata/Hong Kong/2009/JX985686 | JX985686 | 411 | - | 411 | - | 2009-11-27 | China: Hong Kong | Anas clypeata | MPJ0597 |
| Avastrovirus 3 | Avastrovirus 1 isolate MPJ0779/Anas penelope/Hong Kong/2009/JX985688 | JX985688 | 372 | - | 372 | - | 2009-12-17 | China: Hong Kong | Anas penelope | MPJ0779 |
| Avastrovirus 3 | Avastrovirus 1 isolate MPJ1601/Anas acuta/Hong Kong/2010/JX985721 | JX985721 | 396 | - | 396 | - | 2010-01-12 | China: Hong Kong | Anas acuta | MPJ1601 |
| Avastrovirus 3 | Avastrovirus 1 isolate MPK514/Anas crecca/Hong Kong/2009/JX985722 | JX985722 | 405 | - | 405 | - | 2009-12-23 | China: Hong Kong | Anas crecca | MPK514 |
| Avastrovirus 3 | Avastrovirus 1 isolate Sweden 721/Anas platyrhynchos/Sweden Uppsala/2014/KY320413 | KY320413 | 391 | - | 391 | - | 2014-11-17 | Sweden | Anas platyrhynchos | Sweden 721 |
| Avastrovirus 3 | Chicken astrovirus/USA/2007/JF414802 | JF414802 | 7539 | 3420 | 1560 | 2232 | 2007-08 | USA | Chicken | GA2011 |
| Avastrovirus 3 | Duck astrovirus/China/2008/NC_012437 | NC_012437 | 7722 | 3723 | 1551 | 2196 | 2008-07-09 | China | Duck | C-NGB |
| Avastrovirus 3 | Goose astrovirus/China/2014/NC_034567 | NC_034567 | 7299 | 3282 | 1545 | 2124 | 2014-06-04 | China | Hortobagy goose | FLX |
| Avastrovirus 3 | Turkey astrovirus 2/NC_005790 | NC_005790 | 7355 | 3376 | 1541 | 2175 | 2000 | - | Turkey | - |
| Avastrovirus 4 | Avastrovirus 3 isolate MPJ0127/Anas crecca/Hong Kong/2009/JX985680 | JX985680 | 363 | - | 363 | - | 2009-11-06 | China: Hong Kong | Anas crecca | MPJ0127 |
| Avastrovirus 4 | Avastrovirus 3 isolate MPJ0552/Anas clypeata/Hong Kong/2009/JX985682 | JX985682 | 381 | - | 381 | - | 2009-11-27 | China: Hong Kong | Anas clypeata | MPJ0552 |
| Avastrovirus 4 | Avastrovirus 3 isolate MPJ1364/Anas penelope/Hong Kong/2009/JX985709 | JX985709 | 390 | - | 390 | - | 2009-12-23 | China: Hong Kong | Anas penelope | MPJ1364 |
| Avastrovirus 4 | Avastrovirus 3 isolate MPJ1442/Anas acuta/Hong Kong/2009/JX985715 | JX985715 | 372 | - | 372 | - | 2009-12-30 | China: Hong Kong | Anas acuta | MPJ1442 |
| Avastrovirus 4 | Avastrovirus 3 isolate Sweden 701/Anas platyrhynchos/Sweden Uppsala/2014/KY320411 | KY320411 | 376 | - | 376 | - | 2014-10-13 | Sweden | Anas platyrhynchos | Sweden 701 |
| Avastrovirus 5 | Passerine astrovirus 1/French Guiana/2016/MK096773 | MK096773 | 6745 | 2724 | 1578 | 2265 | 2016-10 | French Guiana | Passeriformes | PasAstV-1 |
| Avastrovirus 5 | Passerine astrovirus 2/French Guiana/2016/MK096774 | MK096774 | 6864 | 2706 | 1440 | 2301 | 2016-10 | French Guiana | Passeriformes | PasAstV-2 |
| Avastrovirus 5 | Passerine astrovirus 3/French Guiana/2016/MK096775 | MK096775 | 6628 | 2643 | 1470 | 2256 | 2016-10 | French Guiana | Passeriformes | PasAstV-3 |
| Avastrovirus 5 | Passerine astrovirus 4/French Guiana/2016/MK096776 | MK096776 | 6870 | 2781 | 1209 | 2367 | 2016-10 | French Guiana | Passeriformes | PasAstV-4 |
| Unclassified Astrovirus | Beihai astro-like virus/NC_032439 | NC_032439 | 6856 | 2655 | 1176 | 1893 | 2014 | China | Penaeid shrimp | BHWZXX13371 |
| Unclassified Astrovirus | Guangdong chinese water snake astrovirus/China/MG599904 | MG599904 | 6919 | 2533 | 1791 | 2412 | 2018 | China | Myrrophis chinensis | LPSF9635 |
| Unclassified Astrovirus | Wenling righteye flounders astrovirus/China/MG599899 | MG599899 | 6337 | 3357 | 1536 | 1288 | 2018 | China | Pleuronichthys japonicus | XYHYG25184 |


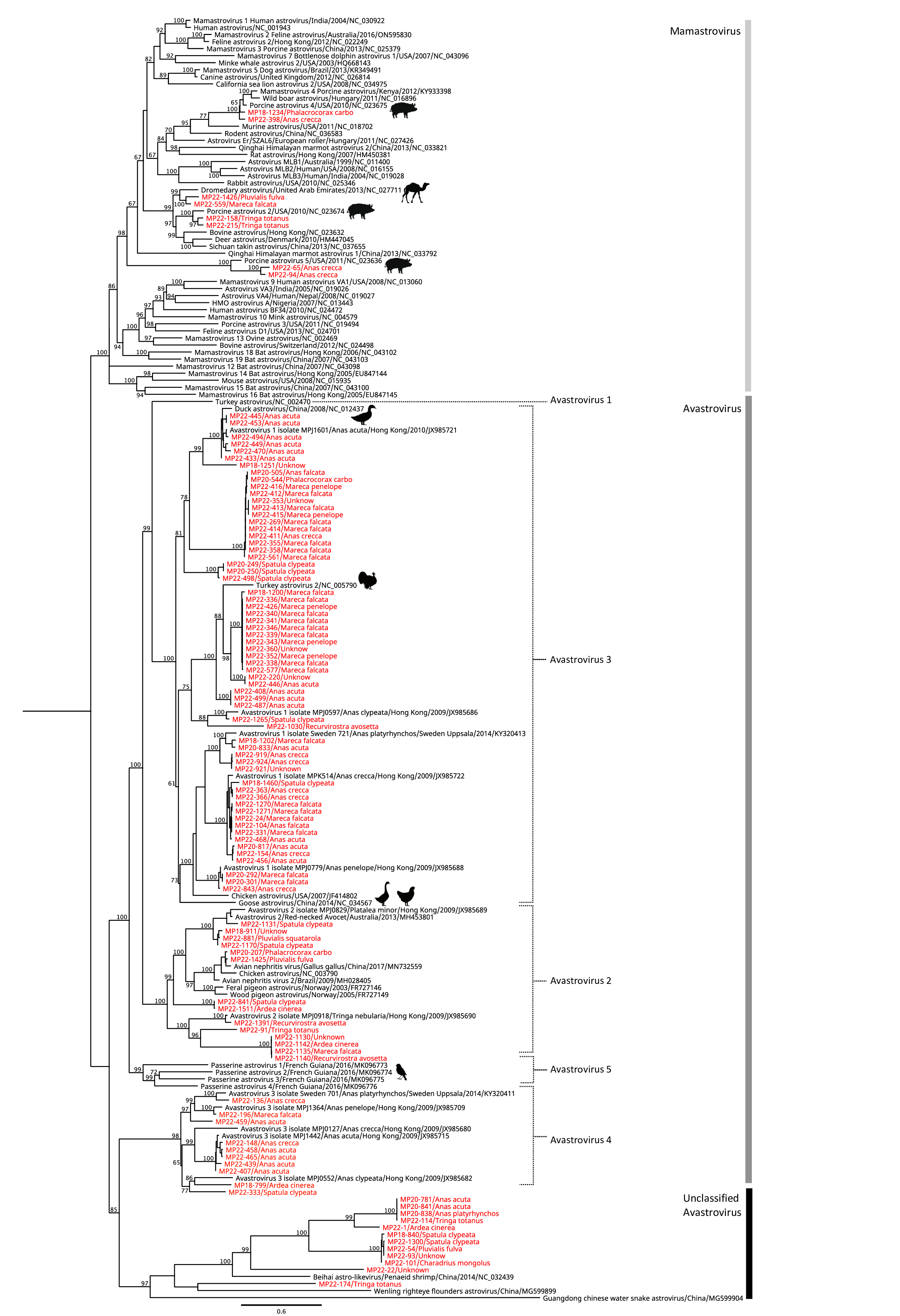


**Figure S1.** Phylogenetic analysis on RdRp region (360bp) of astroviruses using IQ‐TREE by maximum likelihood. The tree was rooted by *Mamastrovirus* group. The branch values are bootstrap supports (%). Bootstrap values were set up with 1000 replicates as statistical support. Astroviruses detected from this study (n = 106) were highlighted in red color and named with sample number, followed by identical host species. Samples showed related to same groups have been indicated by isosceles triangles figure and the total number of sequences in each group is shown next to the triangles. GenBank accession numbers of retrieved genes are indicated in the end of reference name. *Mamastrovirus* group included from *Mamastrovirus* 1 to 19. *Avastrovirus* strains are marked according to the ICTV classification (*Avastrovirus* 1, 2, 3) and our proposed new classification. Three group of *Mamastrovirus*, *Avastrovirus* and unclassified Astrovirus were indicated at the right side of this figure.

| **Table S2.** Selected samples (n=18) including the *Mamastrovirus* group, *Avastrovirus* 4, and unclassified astroviruses for metagenomic analysis. | | | | |
| --- | --- | --- | --- | --- |
| Clades | Sample No. | | | |
| *Mamastrovirus* | MP18-1234 | MP22-65 | MP22-94 | MP22-158 |
|  | MP22-215 | MP22-398 | MP22-559 | MP22-1426 |
| *Avastrovirus* 4 | MP18-799* | MP22-196* | MP22-333 | MP22-439 |
| Unclassified Astroviruses | MP20-841 | MP22-22 | MP22-101 | MP22-114* |
|  | MP22-174 | MP22-1300 |  |  |

* Sample were identified some astrovirus-related contigs.
